# Platelet-derived TGF-β1 is related to portal vein thrombosis in cirrhosis by promoting hypercoagulability and endothelial dysfunction

**DOI:** 10.3389/fcvm.2022.938397

**Published:** 2022-09-26

**Authors:** Siyu Jiang, Yingjie Ai, Liyuan Ni, Ling Wu, Xiaoquan Huang, Shiyao Chen

**Affiliations:** ^1^Department of Gastroenterology and Hepatology, Zhongshan Hospital, Fudan University, Shanghai, China; ^2^Endoscopy Center and Endoscopy Research Institute, Zhongshan Hospital of Fudan University, Shanghai, China

**Keywords:** portal vein thrombosis, transforming growth factor-beta, cirrhosis, endothelial damage, hypercoagulability

## Abstract

**Background:**

Portal vein thrombosis (PVT) is a serious complication of cirrhosis accompanied by unclear pathogenesis. Transforming growth factor-beta (TGF-β) has been implicated in atherosclerosis and venous thrombosis whereas study regarding its part in PVT is lacking. The aim of this study was to explore the role of cytokine TGF-β1 in PVT and the potential mechanism.

**Materials and methods:**

We included patients with cirrhotic gastroesophageal varices and divided them into two groups according to the presence of PVT. Serum levels of TGF-β1 were detected using Cytometric Bead Array kit and compared between two groups. Coagulation status was assessed using thromboelastography (TEG). Primary liver sinusoidal endothelial cells were treated with TGF-β1 and evaluated for endothelial dysfunction by RT-PCR.

**Results:**

Our results uncovered that TGF-β1 (6,866.55 vs. 3,840.60 pg/ml, *P* = 0.015) significantly increased in the PVT group. Splenectomy might promote PVT by increasing platelet-derived TGF-β1 levels. Other cytokines showed no difference between PVT and non-PVT groups. Besides, TGF-β1 was correlated with platelet, fibrinogen, TEG-CI, TEG-MA, and TEG-α (coef = 0.733, 0.494, 0.604, 0.608, and 0.511; *P* < 0.001, 0.027, 0.004, 0.004, and 0.021, respectively), which indicated a hypercoagulable state in PVT patients. RT-PCR of liver sinusoidal endothelial cells showed a markable increment of von Willebrand Factor (vWF), thrombomodulin(TM), intercellular adhesion moleclar-1(ICAM-1), and vascular endothelial growth factor(VEGF) after TGF-β1 treatment, suggesting the involvement of endothelial dysfunction.

**Conclusion:**

Elevated platelet-derived TGF-β1 exhibited association with hypercoagulability and promoting effect on endothelial dysfunction, closely related with PVT in cirrhotic patients.

## Introduction

Portal vein thrombosis (PVT) is a serious complication of cirrhosis accompanied with a poor prognosis (1). The pathogenesis of PVT is multifactorial and components of Virchow’s triad (decreased blood flow, endothelial injury, and hypercoagulability) have been thought to play a key role in the onset and development of PVT (2). Although several studies believed that alteration in portal vein flow plays a predominate part in the development of PVT, the function of endothelial damage in the natural course of PVT cannot be overestimated (1, 3, 4). The underlying mechanism of PVT advancement remains unclear and desiderates further investigations.

Inflammation is believed to have crucial effects on thrombi formation as systemic inflammation has been proved to be associated with increased PVT risk (5, 6). Transforming growth factor-beta (TGF-β), a multifunctional cytokine mainly derived from platelets (7, 8), participates in many biological and pathologic functions, including the immune response, angiogenesis, venous thrombosis, and tissue fibrosis (9). As for endothelial damage, TGF-β is associated with apoptosis of epithelial cells induced by continuous stretch (10). Meanwhile, it can also aggravate venous thrombosis by impairing thrombus resolution and promoting endothelial dysfunction without affecting platelet function (11). Despite that TGF-β has been reported to take part in atherosclerosis and venous thrombosis, there is a lack of studies regarding its role in PVT.

The aim of this study was to investigate the role and potential mechanisms of inflammatory cytokine TGF-β1 promoting PVT through clinical and experimental studies.

## Materials and methods

### Study population

The study was a cross-sectional case-control study to investigate the role of cytokines. Fifty-three patients who had cirrhotic gastroesophageal varices confirmed by CT angiography (CTA) and gastroscopy were enrolled between 1 December 2016, and 31 December 2017. All patients had a history of gastroesophageal variceal hemorrhage. Exclusion criteria were: (1) active bleeding; (2) combination with malignancy; (3) overt infection or sepsis; (4) treatment with systemic antibiotic or anti-inflammatory drugs in the past 2 weeks; (5) severe abdominal pain; (6) coagulation disorders.

All patients were divided into two groups according to the presence of PVT (PVT group, *n* = 20; non-PVT group, *n* = 33). None of them received anticoagulation or antiplatelet therapy before. The protocol for conducting the current analysis was approved by the ethic committee of Zhongshan Hospital, Fudan University (B2015-133R), in accordance with the Declaration of Helsinki.

### Laboratory methods

Measurements were ascertained while blinded to the sample origin. Routine biochemical tests were carried out using standard procedures. Patients had fasted for the last 12 h before blood sampling and underwent routine biochemical evaluations. Serum TGF-β1 (cat. no. 560429), L-selectin (cat. no. 560420), intercellular adhesion moleclar-1 (ICAM-1, cat. no. 560269), and vascular cell adhesion molecule-1 (VCAM-1, cat. no. 560427) were determined using Cytometric Bead Array kit (CBA; BD Biosciences, San Jose, CA, United States). Serum samples were mixed to capture specific beads for each cytokine. Antibodies were added, conjugated with phycoerythrin, and incubated for 2 h under room temperature, protected from light. Tubes were then centrifuged (200 g for 5 min) and the supernatant was carefully aspired and discarded. The pellets containing beads were resuspended and the samples were analyzed on the Thermo Cytometer. The data obtained were analyzed by the BD™ Cell Quest and FCAP Array software.

### Cell culture and treatment

Liver sinusoidal endothelial cells (LSECs, Sciencell, San Diego, California, USA), were cultured in the endothelial cell medium (ECM, 1001, Sciencell, San Diego, California, USA) supplemented with 5% fetal bovine serum and 1% penicillin–streptomycin. Cells were incubated in humidified atmosphere of 5% CO_2_ at 37°C.

After seeding, LSEC cells were stimulated with different concentrations of TGF-β1 (0, 1, 5, 10, 50, 100 ng/mL, 100-21, PeproTech, Cranbury, New Jersey, USA) for 48 h. The cells were collected for real-time PCR to measure the effects of TGF-β1 on endothelial cells.

### Reverse transcription-polymerase chain reaction

The cellular RNA was extracted by TRIzol (15596018, Thermo Fisher Scientific, Carlsbad, California, USA) and reverse-transcribed into cDNA using a commercial reverse transcription kit (A5000, Promega, Beijing, China). The real-time PCR was performed using a SYBR Green PCR Master Mix (FP215, Tiangen, Beijing, China) in accordance with the manufacturer’s protocol. Target genes were quantified using the 2^–ΔΔ^*^Ct^* method and normalization with the expression of *GAPDH*.

The primers used were as follows:

**Table:** 

Gene	Species	Forward 5′ →3′	Reverse 5′→3′
*thrombomodulin (TM)*	Homo	CGACCTTCCT CAATGCCAGT CAG	CGTCGCCGTT CAGTAGCAAGG
*vWF*	Homo	GAAGCAGACG ATGGTGGATTC CTC	AGCAATGGT GTCGCAGAA GCAG
*ICAM-1*	Homo	GTCACCTATGGC AACGACTCCTTC	AGTGTCTCCT GGCTCTGG TTCC
*VEGF*	Homo	AGAAGGAGGAG GGCAGAATCAT CAC	GGGCACACAGG ATGGCTTGAAG

### Thromboelastography

Thromboelastography (TEG) (TEG 5000, Thrombelastograph Hemostasis Analyzer System, Haemonetics Corporation, Braintree, MA, USA) was performed to assess coagulation profile. Clot formation was triggered using Kaolin *in vitro*. The variables of TEG include rate of clot formation (α angle), maximum amplitude (MA), clot time (R), clot formation time (K), and coagulation index (CI).

### Statistics

Continuous variable data were reported as mean ± standard error (when the values were normally distributed) or median and interquartile range (IQR) (when the values had a distribution that departed significantly from normal). All data regarding categorical variables are shown as *n* (proportion). We tested the independence of categorical variables by *x*^2^ test. We used Student’ unpaired *t*-test and Pearson correlation analysis to evaluate normally distributed continuous variables. Mann–Whitney U test was employed for all the other variables between the two groups. Levene’s test was used for testing the equality of variances. Pearson correlation analysis were performed to explore the relationship between TGF-β1 and factors associated with coagulation and endothelial injury. Only two-tailed probabilities were used for testing statistical significance. Probability values < 0.05 were regarded as statistically significant. All calculations were made with the SPSS 24.0.

## Results

### General characteristics

A total of 53 patients with cirrhotic varices were included and the characteristics of all individuals were summarized in Table 1. The baseline characteristics indicated that males accounted for 43.40% and the mean age of included individuals was 54 ± 11 years old. Of 53 patients, there were 6 (13.04%) participants who had hypertension and 23 (43.40%) diabetes. A total of 17 (36.17%) patients had experienced splenectomy. Most patients were Child-Pugh A (*n* = 32, 60.38%) while 21 (39.62%) patients were Child-Pugh B. About half of the included patients suffered from hepatitis B cirrhosis (54.72%).

**TABLE 1:** Demographic characteristics and laboratory exanimations of cirrhotic patients.

	All patients (*n* = 53)	PVT (*n* = 20)	Non-PVT (*n* = 33)	*P*-value
Age (years)	53.66 ± 11.27	58.15 ± 11.38	50.94 ± 10.45	**0.027**
Gender (male%)	23(43.40%)	12(60.00%)	11(33.33%)	0.107
Splenectomy (%)	17(36.17%)	12(63.16%)	5(17.86%)	**0.004**
Hypertension (%)	6(13.04%)	5(26.32%)	1(3.70%)	0.072
Diabetes (%)	23(43.40%)	12(60.00%)	11(33.33%)	0.107
HVPG (mmHg)	18.52 ± 3.42	17.18 ± 2.86	19.32 ± 3.51	**0.020**
Child-Pugh (A/B/C)(%)				0.361
A	32(60.38%)	10(50.00%)	22(66.67%)	
B	21(39.62%)	10(50.00%)	11(33.33%)	
C	0	0	0	
Pathogeny (%)				0.411
Hepatitis B infection	29(54.72%)	9(45.00%)	20(60.61%)	
Other causes	24(45.28%)	11(55.00%)	13(39.39%)	
Platelet (×10^9^/L)	64.00(48.00–134.00)	127.50(56.75–220.50)	60.00(45.00–80.00)	**0.007**
ALT (U/L)	18.00(13.00–30.00)	15.50(11.75–19.12)	22.00(15.00–32.00)	0.130
Total bilirubin (μmol/L)	13.90(10.60–17.00)	14.45(11.10–19.05)	13.40(10.60–16.50)	0.399
Albumin (g/L)	36.53 ± 4.87	33.98 ± 4.54	38.08 ± 4.44	**0.003**
D-dimer (mg/L)	0.76(0.33–1.86)	1.50(1.00–4.04)	0.36(0.28–1.06)	**<0.001**
CRP (mg/L)	1.10(0.50–3.60)	1.80(0.80–5.10)	1.07(0.30–2.30)	0.107
Prothrombin time (s)	19.00(18.20–19.90)	19.35(18.20–20.25)	18.60(18.10–19.60)	0.205
APTT(s)	28.50(26.00–30.20)	28.45(26.30–30.65)	28.70(26.00–29.90)	0.727

Significant values are expressed in bold.

Patients were divided into the PVT and non-PVT groups (*n* = 20 vs. 33). There was no significant difference observed in gender, hypertension, diabetes, or cirrhosis etiology between the two groups. More patients in the PVT group had experienced splenectomy (12 vs. 5, *P* = 0.004). Platelet counts significantly increased in the PVT group (127.50 vs. 60.00 × 10^9/*L*^, *P* = 0.007) as albumin(33.98 ± 4.54 vs. 38.08 ± 4.44 g/L, *P* = 0.003) remarkably decreased. Correspondingly, ALT, total bilirubin, prothrombin time, and APTT showed no significant difference.

### Transforming growth factor-beta1 was related to the occurrence of portal vein thrombosis in cirrhosis

The comparison of inflammatory mediators was performed in cirrhotic patients with or without PVT. As shown in Table 2, TGF-β1 [8.78 ± 0.71 vs. 8.25 ± 0.64 log (pg/ml), *P* = 0.009] interleukin 6 (IL-6, 4.80 vs. 2.40, *P* = 0.045) significantly increased in the PVT group, whereas other inflammatory mediators [procalcitonin (PCT), C-reactive protein (CRP), lipopolysaccharides (LPS), erythrocyte sedimentation rate (ESR), tumor necrosis factorα (TNFα), interleukin 2 receptor (IL-2R), interleukin 6 (IL-6), and interleukin 8 (IL-8)] showed no difference between two groups. After being stratified by splenectomy, no significant difference in TGF-β1 was observed between the two groups [8.26 ± 0.68 vs. 8.12 ± 0.59, *P* = 0.611 log (pg/ml); 9.06 ± 0.60 vs. 8.77 ± 0.84 log (pg/ml), *P* = 0.515 in non-splenectomized and splenectomized patients, respectively] (Supplementary Tables 1, 2). Besides, we found that patients with splenectomy exhibited significantly increased platelets (166.82 ± 94.67 vs. 73.95 ± 55.27 × 10^9^, *P* = 0.001) and TGF-β1 levels [8.98 ± 0.66 vs. 8.15 ± 0.60 log (pg/ml), *P* < 0.001] compared with those without splenectomy.

**TABLE 2:** The comparison of levels of inflammatory cytokines between portal vein thrombosis (PVT) and non-PVT groups.

Characteristics	All patients (*n* = 53)	PVT (*n* = 20)	non-PVT (*n* = 33)	*P*-value
PCT(μg/L)	0.05 (0.03–0.08)	0.06 (0.03–0.10)	0.05 (0.03–0.07)	0.619
CRP (mg/L)	1.10 (0.50–3.60)	1.80 (0.80–5.10)	1.07 (0.30–2.30)	0.107
LPS(pg/ml)	0.03 (0.02–0.04)	0.03 (0.02–0.04)	0.02 (0.02–0.04)	0.673
ESR(mm/H)	13.00 (6.00–24.00)	16.00 (6.00–22.50)	12.00 (5.00–27.00)	0.692
TGF-β1 (pg/ml)	4598.70 (2844.60–7355.40)	6866.55 (3492.08–13281.68)	3840.60 (2518.20–5163.00)	**0.015**
log(TGF-β1)	8.45 ± 0.71	8.78 ± 0.71	8.25 ± 0.64	**0.009**
TNFα(pg/ml)	11.30 (8.89–18.90)	11.95 (9.38–29.28)	10.80 (8.20–18.90)	0.229
IL-2R(U/ml)	434.00 (341.00–532.00)	414.50 (365.00–487.25)	434.00 (326.00–532.00)	0.949
IL-6(pg/ml)	3.20 (2.14–6.44)	4.80 (2.39–8.68)	2.40 (2.10–5.30)	**0.045**
IL-8(pg/ml)	17.40 (6.10–44.60)	14.80 (7.08–58.68)	18.90 (5.60–38.50)	0.613

*Significant values are expressed in bold.

To investigate the relationship between cirrhosis and TGF-β1, the levels of inflammatory mediators were compared between healthy individuals and cirrhotic patients. A slight increase tendency of TGF-b1 levels (8.45 ± 0.71 vs. 8.56 ± 0.17 log(pg/ml), *P* = 0.303) was observed in cirrhosis (Supplementary Table 3).

**FIGURE 1:**
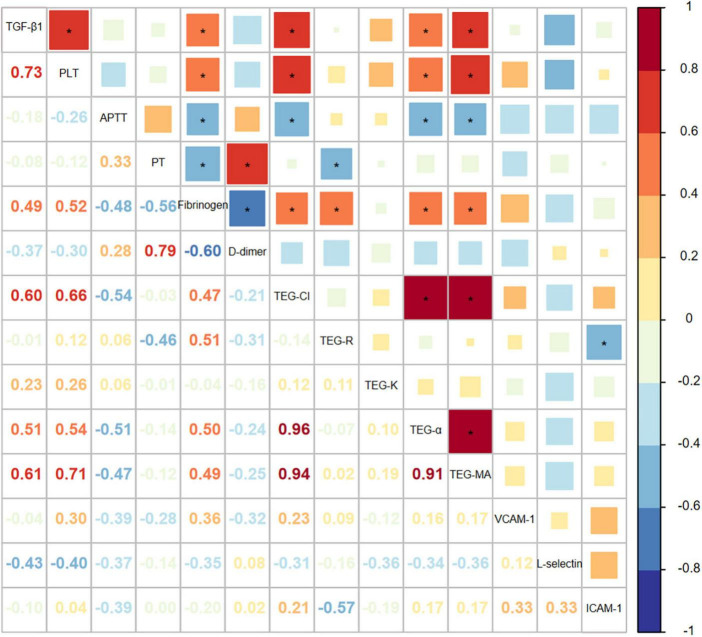
The correlation between TGF-β1 and factors associated with coagulation and endothelial dysfunction in portal vein thrombosis (PVT) patients.

To ascertain whether a change of TGF-β1 influences the pathogenesis of PVT, the correlation between TGF-β1 and parameters associated with coagulation and endothelial dysfunction were explored (Figure 1). The Pearson’s correlation coefficient between TGF-β1 and platelet as well as fibrinogen was 0.733 (*P* < 0.001) and 0.494 (*P* = 0.027), respectively, which is correspondent with TGF-β1’s derivation from platelets. TEG showed that TEG-CI (*P* = 0.004), TEG-MA (*P* = 0.004), and TEG-α (*P* = 0.021) were closely related to TGF-β1 as Pearson’s correlation coefficients were 0.604, 0.608, and 0.511, respectively. TEG-MA represents the interaction between platelet and fibrinogen, and TEG-α represents fibrinogen levels, reflecting the strength and rate of clot formation. Taken together, the relationships between TGF-β1 and fibrinogen and parameters of TEG indicate that TGF-β1 may partake in the hypercoagulable status in PVT.

### Transforming growth factor-beta1 contributes to endothelial dysfunction

**FIGURE 2:**
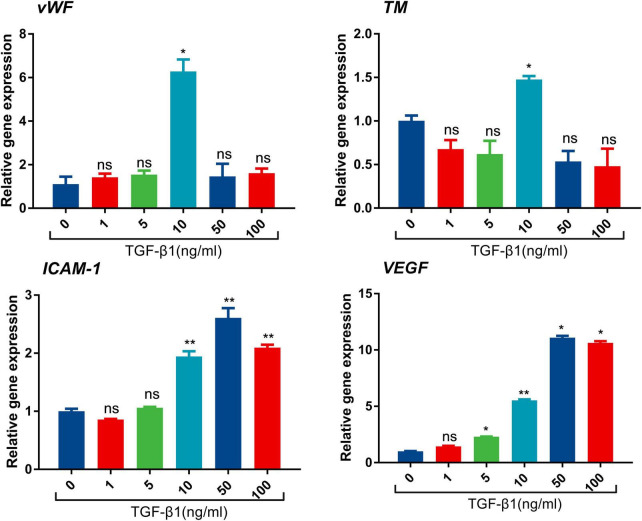
mRNA expression of vWF, intercellular adhesion moleclar-1 (ICAM-1), TM, and VEGF after the treatment of TGF-β1 in endothelial cells. (*p < 0.05, **p < 0.01). Data are expressed as mean ± SEM. Three replicates per group.

To further investigate the influence of TGF-β1 on endothelial cells, *in vitro* experiments were subsequently performed. LSECs were cultured and treated with different concentrations of TGF-β1. The mRNA expression of endothelial damage [von Willebrand Factor (vWF), thrombomodulin (TM)], adhesion molecule (ICAM-1), and angiogenesis vascular endothelial growth factor (VEGF) was significantly increased after the treatment of TGF-β1 (Figure 2). The alterations of ICAM-1, and VEGF mRNA expression were in parallel with the concentration of TGF-β1. These results provided mechanistic evidence for pro-thrombotic effects of TGF-β1 in PVT.

## Discussion

In this study, we discovered that TGF-β1 was significantly elevated in cirrhotic patients with PVT. The results suggested that TGF-β1 was positively correlated with platelet and fibrinogen, TEG-CI, TEG-MA, as well as TEG-α, which uncovered a hypercoagulable state. *In vitro* experiments verified the pro-thrombotic effects of TGF-β1 through up-regulating vWF, TM, ICAM-1, and VEGF. Our study showed that TGF-β1 plays a pivotal role in PVT by partaking in hypercoagulability and promoting endothelial dysfunction in cirrhosis.

As a common and serious complication of cirrhosis, the clinical impact of PVT varies from asymptomatic to life-threatening conditions, which is related to accumulating risk of variceal bleeding and mortality (12–15). The mechanism of cirrhosis-induced PVT is multifactorial. Previous studies mainly focused on Virchow’s triad, but further investigations regarding the role of the inflammatory mediator, TGF-β1, in PVT development are lacking. Anticoagulation therapy is a well-accepted therapeutic method for PVT. However, the efficacy of anticoagulation treatment is far from ideal, as only 42–53% of cases could achieve complete portal vein recanalization (16). Besides, some patients exhibited poor responses to anticoagulation treatment in clinical practice (16, 17). The unsatisfactory performance of anticoagulation treatment suggested that PVT might have other underlying mechanisms, which deserves further investigation.

Accumulating evidence indicated that inflammatory response could simultaneously act as a cause and consequence of venous thrombosis (18). Systemic and local inflammation contributes to the occurrence of PVT (5, 19–21). TGF-β1 family is composed of many multifunctional cytokines, including TGF-β1, bone morphogenetic proteins and anti-Müllerian hormone (22).

Previous studies have explored the role of TGF-β1 in bleeding events and coagulation. TGF-β1 polymorphisms could result in vascular malformations, which are associated with an increased risk of bleeding complications. Kim (23) proposed that TGF-β1 polymorphisms were associated with bleeding complications in patients with warfarin therapy after cardiac valve replacement. For patients with cirrhotic portal hypertension, TGF-β1 plays a prominent role in angiogenesis and vascular development, resulting in the formation of collateral vessels. As a result of that, elevated TGF-β1 in cirrhosis may not increase the risk of bleeding. The relationships between TGF-β1 and hemorrhage in cirrhosis need further investigations. Numerous researches have been carried out on the effects of TGF-β1 on atherosclerosis and venous thrombosis, but little attention has been paid to the role of TGF-β1 in PVT development. The effects of TGF-β1 are controversial in atherosclerosis. On the one hand, TGF-β1 was believed to exert antiatherogenic effects (24) and on the other hand, some studies proposed that endothelial-derived TGF-β1 lead to vessel wall inflammation and atherosclerotic plaque growth (25–27). In venous thrombosis, the knockout of TGF-β1 resulted in smaller thrombi in mice without affecting platelet function. TGF β1 signaling *via* TGF-β1RI impairs thrombus resolution by favoring fibrosis and endothelial dysfunction (11). Our previous study has confirmed that continuous stretch of hepatic endothelial cells leads to upregulation of TGF-β1, caused by increased ROS levels (10). Lee (28) found a significant increase in plasma TGF-β1 levels in hepatocellular carcinoma (HCC) patients with PVT compared with those without tumoral PVT. It is difficult to distinguish blood clot formation in the portal venous system from tumoral PVT but their mechanisms are completely different (1). In order to investigate the effects of TGF-β1 on blood clot formation in cirrhotic PVT, patients with hepatic carcinoma and other malignancy were excluded. Our results elucidate that elevated TGF-β1 is closely associated with non-tumoral PVT. As it is widely believed that 40–45% of plasma TGF-β1 was secreted by platelets, the relationship between TGF-β1 and platelets was consistent with our results, suggesting the potent potential value of TGF-β1 in PVT development.

Splenectomy is the independent risk factor of PVT and significantly contributes to the increase of platelets. At the same time, platelets are the leading carrier of TGFβ in the body, responsible for about 40–45% of all TGF-β1 in peripheral blood. According to our results, a relatively higher prevalence of splenectomy was observed in the PVT group and patients with splenectomy had higher platelet levels and almost the same TGF-β1 levels in the PVT group. The absence of association between TGF-β1 and PVT after stratifying splenectomy indicated that splenectomy had a pivotal effect on PVT onset. Previous studies have elucidated increased TGF-β1 production after splenectomy, inconsistent with our results (29, 30). Besides, platelets and TGF-β1 exhibited a strong positive correlation (coef: 0.733, *P* < 0.001) based on our findings. Therefore, we supposed that elevated TGF-β1 levels derived from platelets might be owing to splenectomy. Splenectomy may promote PVT development by upregulating TGF-β1 levels in cirrhosis.

During blood clotting, various agents, such as thrombin, could activate platelet and thus lead to the secretion of TGF-β1 (31). The close association between TGF-β1 and fibrinogen as well as parameters of TEG in our results corroborated that TGF-β1 was related to activated coagulation process in the course of blood clotting. The augment in markers of endothelial injury, adhesion molecules, and angiogenesis indicated endothelial dysfunction and reflected a thrombotic tendency. RT-PCR confirmed the prothrombotic effects of TGF-β1 by promoting endothelial dysfunction. Our investigation of the mechanisms of TGF-β1 in PVT helps gain greater insight into molecular pathogenesis. The pathogenetic effects of TGF-β1 in promoting thrombosis deserve further investigation.

This study has a few limitations. First of all, this is a cross-sectional study so only the association between TGF-β1 and PVT could be explored. Secondly, only reverse transcription-polymerase chain reaction (RT-PCR) was performed to evaluate the function of TGF-β1 on endothelial cells. Protein assessment and other molecular biology experiments are needed. Moreover, larger prospective validation studies, as well as mechanistic studies, should be conducted in the future.

## Conclusion

Transforming growth factor-1 was related to PVT in cirrhotic patients, which was closely associated with hypercoagulable status and promoting hepatic endothelial dysfunction. Further clinical and mechanistic studies are needed to investigate the role of TGF-β1 as a potential pathogenetic factor of PVT in the cirrhotic population.

## Data availability statement

The raw data supporting the conclusions of this article will be made available by the authors, without undue reservation.

## Ethics statement

The studies involving human participants were reviewed and approved by the Ethic Committee of Zhongshan Hospital, Fudan University. The patients/participants provided their written informed consent to participate in this study.

## Author contributions

SJ and YA performed the experiments, analyzed, interpreted the data, and drafted the manuscript. LN and LW helped in data collection and data analysis. XH conceived the study, collected, and performed the critical revision of the manuscript. SC contributed significantly to critical revision of the manuscript. All authors contributed to the study and involved in critical revision of the manuscript for important intellectual content.

## Abbreviations

PVT: portal vein thrombosis
TGF- β: transforming growth factor-beta
LSECs: liver sinusoidal endothelial cells
ICAM-1: intercellular adhesion moleclar-1
VCAM-1: vascular cell adhesion molecule-1
TEG: thromboelastography
PCT: procalcitonin
CRP: C-reactive protein
LPS: lipopolysaccharides
ESR: erythrocyte sedimentation rate
TNF α: tumor necrosis factor α
IL-2R: interleukin 2 receptor
IL-6: interleukin 6
IL-8: interleukin 8.
