# Supplementary material for: Platelet-derived TGF-β1 is related to portal vein thrombosis in cirrhosis by promoting hypercoagulability and endothelial dysfunction
Source: Front Cardiovasc Med. 2022 Sep 26;9:938397. doi: 10.3389/fcvm.2022.938397 (PMC9548594; doi:10.3389/fcvm.2022.938397)
Supplement: Supplementary file 1 [file Data_Sheet_1.docx]

**Supplementary Table 1. The comparison of TGF-β1 levels between PVT and non-PVT groups in non-splenectomized patients**

|  | Non-splenectomized patients(n=30) | PVT(n=7) | non-PVT(n=23) | P value |
| --- | --- | --- | --- | --- |
| TGF-β1 (pg/ml) | 3536.40(2457.68-4732.88) | 3284.40(2490.00-4775.55) | 3728.10(2477.85-4713.75) | 0.961 |
| log(TGF-β1 ) | 8.15±0.60 | 8.26±0.68 | 8.12±0.59 | 0.611 |

**Supplementary Table 2. The comparison of TGF-β1 levels between PVT and non-PVT groups in splenectomized patients**

|  | splenectomized patients(n=17) | PVT(n=12) | non-PVT(n=5) | P value |
| --- | --- | --- | --- | --- |
| TGF-β1 (pg/ml) | 7944.60(6144.90-14022.60) | 7971.60(6504.00-14356.95) | 7355.40(5163.00-11414.70) | 0.506 |
| log(TGF-β1 ) | 8.98±0.66 | 9.06±0.60 | 8.77±0.84 | 0.515 |

**Supplementary Table 3. The comparison of TGF-β1 levels between cirrhotic patients and healthy controls.**

|  | all patients(n=65) | cirrhosis(n=53) | healthy control(n=12) | P value |
| --- | --- | --- | --- | --- |
| TGF-β1 (pg/ml) | 4764.30(3284.40-6576.90) | 4598.70(2844.60-7355.40) | 5376.15(4842.38-5792.33) | 0.357 |
| log(TGF-β1 ) | 8.47±0.65 | 8.45±0.71 | 8.56±0.17 | 0.303 |
